# Mechanism-based genotoxicity screening of metal oxide nanoparticles using the ToxTracker panel of reporter cell lines

**DOI:** 10.1186/s12989-014-0041-9

**Published:** 2014-09-02

**Authors:** Hanna L Karlsson, Anda R Gliga, Fabienne MGR Calléja, Cátia SAG Gonçalves, Inger Odnevall Wallinder, Harry Vrieling, Bengt Fadeel, Giel Hendriks

**Affiliations:** 1 Nanosafety & Nanomedicine Laboratory, Division of Molecular Toxicology, Institute of Environmental Medicine, Karolinska Institutet, Stockholm, Sweden; 2 Department of Toxicogenetics, Leiden University Medical Center, Leiden, the Netherlands; 3 KTH Royal Institute of Technology, Division of Surface and Corrosion Science, School of Chemical Science and Engineering, Stockholm, Sweden

**Keywords:** Metal oxide nanoparticles, Nanomaterials, ToxTracker, High-throughput screening, Genotoxicity, Oxidative stress, Reporter cells

## Abstract

**Background:**

The rapid expansion of manufacturing and use of nano-sized materials fuels the demand for fast and reliable assays to identify their potential hazardous properties and underlying mechanisms. The ToxTracker assay is a recently developed mechanism-based reporter assay based on mouse embryonic stem (mES) cells that uses GFP-tagged biomarkers for detection of DNA damage, oxidative stress and general cellular stress upon exposure. Here, we evaluated the ability of the ToxTracker assay to identify the hazardous properties and underlying mechanisms of a panel of metal oxide- and silver nanoparticles (NPs) as well as additional non-metallic materials (diesel, carbon nanotubes and quartz).

**Methods:**

The metal oxide- and silver nanoparticles were characterized in terms of agglomeration and ion release in cell medium (using photon cross correlation spectroscopy and inductively coupled plasma with optical emission spectroscopy, respectively) as well as acellular ROS production (DCFH-DA assay). Cellular uptake was investigated by means of transmission electron microscopy. GFP reporter induction and cytotoxicity of the NPs was simultaneously determined using flow cytometry, and genotoxicity was further tested using conventional assays (comet assay, γ-H_2_AX and RAD51 foci formation).

**Results:**

We show that the reporter cells were able to take up nanoparticles and, furthermore, that exposure to CuO, NiO and ZnO nanoparticles as well as to quartz resulted in activation of the oxidative stress reporter, although only at high cytotoxicity for ZnO. NiO NPs activated additionally a p53-associated cellular stress response, indicating additional reactive properties. Conventional assays for genotoxicity assessment confirmed the response observed in the ToxTracker assay. We show for CuO NPs that the induction of oxidative stress is likely the consequence of released Cu ions whereas the effect by NiO was related to the particles *per se*. The DNA replication stress-induced reporter, which is most strongly associated with carcinogenicity, was not activated by any of the tested nanoparticles.

**Conclusions:**

We conclude that the ToxTracker reporter system can be used as a rapid mechanism-based tool for the identification of hazardous properties of metal oxide NPs. Furthermore, genotoxicity of metal oxide NPs seems to occur mainly via oxidative stress rather than direct DNA binding with subsequent replication stress.

## Background

The exponential increase in the total number of engineered nanoparticles (NPs) for research, development, and commercialization requires tools for rapid and efficient toxicity screening [1]. Ultimately, such rapid screening should allow for mechanistic profiling in order to better inform on hazard identification and to improve risk assessment [2]. Oxidative stress has been identified as a major mechanism of toxicity for nanoparticles. The so-called oxidative stress paradigm describes how increased levels of reactive oxygen species (ROS) lead to various cellular responses, such as antioxidant response, inflammation and cytotoxicity, following nanoparticle-cell interactions [3]. ROS and inflammation can for instance be a consequence of the released toxic metal ions or a reactive particle surface leading to lysosomal destabilization [4],[5]. One main concern following exposure to inhalable particles is their potential genotoxicity, which is closely associated with carcinogenesis. The mechanisms of NP genotoxicity are still not well understood but oxidative stress and/or direct interactions with DNA are considered important [6],[7]. Direct DNA interaction could represent a more nano-specific mechanism due to the fact that small nanoparticles may reach the nucleus via transportation through the nuclear pore complexes [8]. However, also larger nanoparticles of *e.g*. silver (60 nm) [9], SiO_2_ (40–70 nm) [10] and CuO (50–100 nm) [11] have been observed in the nucleus suggesting that larger NPs may get access to the DNA in dividing cells when the nuclear membrane disassembles.

The most commonly used methods for assessing genotoxicity of nanomaterials up to date are the comet assay and the micronucleus (MN) tests [6],[12]. However, such assays are time consuming and give limited information on the mechanisms of damage, thereby hampering human hazard assessment. An alternative approach for rapid genotoxicity testing could be reporter cell systems in which the induction of certain genes can be studied by a simple readout such as luminescence or fluorescence. These reporter assays indicate the cellular signaling pathways that are activated upon exposure, thereby providing insight into the mechanisms of toxicity. However, only very few examples of studies using reporter cell lines for assessing toxicity of NPs have been reported [13]. The mammalian GreenScreen HC assay that uses a Gadd45α-GFP (green fluorescent protein) reporter gene in TK6 human lymphoma cells [14] has been extensively validated for genotoxicity testing of chemicals. Gadd45α is directly controlled by the tumor suppressor p53 but also by various additional cellular stress-related signaling pathways, thereby limiting the usability of the GreenScreen HC assay for identification of the primary mechanism of toxicity of compounds [15].

**Figure 1:**
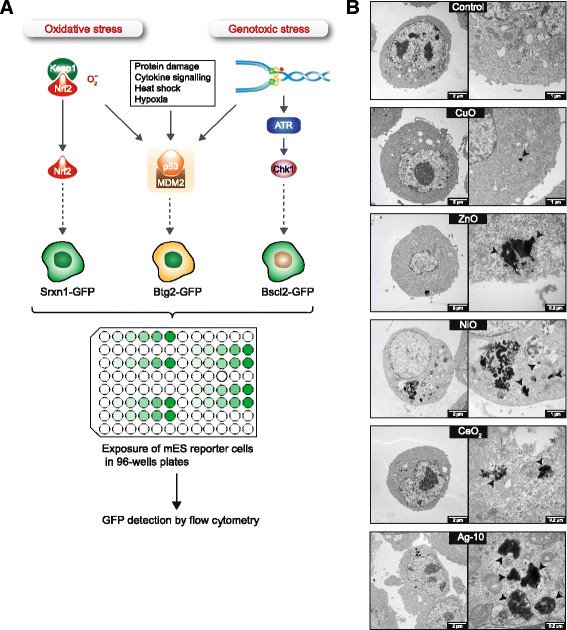
**The ToxTracker reporter assay for mechanism**-**based toxicity testing. (A)** The ToxTracker assay consists of a panel of GFP-based mES cell lines. The GFP reporters indicate activation of the Nrf2-associated antioxidant response, ATR-associated DNA damage response and the p53 cellular stress response. Induction of the GFP reporters as well as cytotoxicity is determined by flow cytometry. **(B)** Cellular uptake but no evidence for nuclear localization of metal oxide NPs in mES cells. Internalization of the NPs after 24 h exposure to 20 μg/mL CuO, 30 μg/mL ZnO, 100 μg/mL NiO, 100 μg/mL CeO_2_ and 10 μg/mL Ag-10 NPs was determined by means of TEM. NPs were taken up by mES cells and were localized in endosomal vesicles or free in the cytoplasm (black arrow heads).

Recently, we developed an *in vitro* assay called ToxTracker that can rapidly provide mechanistic insight into the biological damage induced by chemicals [16]. The ToxTracker assay consists of a panel of mouse embryonic stem (mES) cell lines that each contains a different GFP-tagged reporter for a distinct cellular signaling pathway. The preferential induction of the different reporters indicates the nature of biological damage and associated cellular response pathways. The ToxTracker assay can discriminate between the induction of DNA damage via direct DNA interaction, oxidative stress and general cellular stress (Figure 1A). The DNA damage-associated Bscl2-GFP reporter depends on the ATR (ataxia telangiectasia mutated and Rad3-related)-associated DNA damage signaling pathway and is selectively activated after exposure to genotoxic agents and the subsequent interference with DNA replication [16]. The Srxn1-GFP reporter is preferentially induced upon oxidative stress and is part of the Nrf2 (Nuclear Factor, Erythroid Derived 2, Like 2) antioxidant response pathway. Finally, the Btg2-GFP reporter gene is controlled by p53 and is activated by various types of cellular stress. The combination of different fluorescent reporter cell lines in a single toxicity assay allows not only for rapid and reliable identification of genotoxic properties of chemicals but also enables mechanistic understanding of different modes of toxicity [16].

Here we investigated whether the ToxTracker assay could be used as a rapid mechanism-based tool for assessing genotoxic effects of NPs. In addition, we explored particle *vs*. ion effects in order to identify the particle properties that determine the reporter cell response. For this purpose we used a panel of well-characterized nanomaterials including metal oxide NPs (CuO, ZnO, NiO, CeO_2_, Fe_3_O_4_, TiO_2_) with a primary particle size <100 nm and Ag NPs of two specific sizes (10 nm and 40 nm). Additionally, the quartz material DQ12 was included as a poorly soluble benchmark particle, and the results were compared with diesel particles (standard reference material SRM1650b) and multi-walled carbon nanotubes (MWCNTs). The selection of metal oxide NPs was based on their various abilities to cause DNA damage and oxidative stress upon exposure of lung epithelial cells [17],[18]. Results from the ToxTracker reporter assay were compared with conventional assays for genotoxicity assessment (comet assay, γ-H_2_AX and RAD51 foci formation) and NP uptake was confirmed by transmission electron microscopy (TEM).

## Results

### Characterization of particle solubility, agglomeration and reactivity

Metal oxide NPs (CuO, ZnO, NiO, CeO_2_, Fe_3_O_4_, TiO_2_) and silver NPs of different sizes (10 nm and 40 nm, citrate coated) were carefully characterized in terms of primary size, zeta potential, metal ion release in cell medium, and acellular ROS generation (Table 1). All tested NPs agglomerated to various extents in mES cell culture medium with bimodal and trimodal distributions, but all suspensions contained particles sized <100 nm (see Table 1 and Additional file 1: Figure S1). As expected, the NPs showed a high variation in their ability to dissolve/release metal ions in the cell medium. ZnO NPs dissolved rapidly with approximately 40% reduction in total particle mass already after sonication and centrifugation (0 h), also indicated by a rapid reduction in particle size and scattered light intensity (kcps) as observed using PCCS measurements (data not shown). Only 20% of the particle mass remained in solution after 24 h of exposure (see Table 1). CuO NPs also dissolved to a relatively large extent with time, although significantly slower (2.5% of the total particle mass after 0 h and 37% after 24 h). Similar kinetics were evident for the Ag NPs that showed a clear size-dependent metal release with approximately 22% (Ag-10) and 11% (Ag-40) of the particle mass dissolved after 24 h. The amount of dissolved metals in solution after 24 h was significantly lower for NiO (5% after 24 h), and even lower for TiO_2_ (<0.1%), Fe_3_O_4_ and CeO_2_ (<< 0.01%). NiO NPs were by far the most efficient particles to generate ROS acellularly, followed by CuO (though to a significantly lower extent) whereas the other NPs showed no or only a slight increase in ROS production.

**Table 1:** **Physico**-**chemical characterization of the metal and metal oxide nanoparticles**

**Particle**	**Size**^**a**^**(nm,TEM)**	**Size cell medium(approximate peak max,nm PCCS)**	**Zeta potential,mV**	**Dissolved amount of metals in cell medium,wt%**		**Acellular ROS**^**b**^
				**0 h**	**24 h**	
CuO	20-40	3 and 500	+31	2.5	37.2	2.3
Fe_3_O_4_	20-40	2, 40 and 800	+2	<0.01	<0.01	1.5
ZnO	20-200	20, 500 and 800	+27	41.3	78.5	1.1
TiO_2_	20-100	8 and 400	+6	0.1	0.1	1.3
NiO	2-70	20 and 500	+30	0.9	5.1	>10
CeO_2_	4-30	8 and 80	+4	<0.01	<0.01	1.3
Ag 10	10	6, 40 and 200	N/A	3.0	21.6	0.97
Ag 40	40	10-40 and 200	−41.5	0.2	10.6	0.93

^a^Size, estimated from TEM images, and zeta potential (in MilliQ water with 1 mM NaCl) were analyzed in previous studies (Karlsson *et al*., 2008 [17], Kain *et al*., 2012 [18]), Ag NPs were received from Nanocomposix and were carefully characterized in Gliga *et al*., 2014 [31].

^b^Acellular ROS: Times increase compared to control in ROS kinetics (mean slope per min), 10 μg/mL particle dispersions.

### Cellular uptake, but no evidence for nuclear localization of metal oxide NPs

In order to investigate the general applicability of the recently developed mES cell-based ToxTracker assay for nanoparticle toxicity testing (Figure 1A), we first investigated the ability of the mES cells to internalize NPs by means of TEM and by analyzing the side scatter shift by using flow cytometry. Indeed, NPs may cause genotoxic effects via indirect mechanisms [19], but without cellular uptake the applicability of the assay for NP testing will be limited as in the case for the Ames mutagenicity test [20]. Clearly, all the tested metal and metal oxide NPs analyzed using TEM were internalized by the mES cells, but no nuclear localization was observed (Figure 1B). A significant side scatter shift was also observed for all particles in the study (Additional file 1: Figure S2). Although contributions by possible particles tightly bound to the outer cell membrane cannot be excluded, this data support the uptake observed in the TEM analysis. Fewer internalized particles were observed after exposure to ZnO and CuO NPs due to their extra- and intracellular dissolution. To further confirm uptake of CuO NPs we quantified the metal content after exposure of mES cells to 20 μg/mL for 4 h by means of atomic absorption spectroscopy (AAS). The results showed a metal content of approx. 6.1 pg/cell which is in the same range as our previous studies on CuO NPs uptake in A549 and BEAS-2B cells [21].

### The ToxTracker assay identifies distinct toxicity responses to NPs

**Figure 2:**
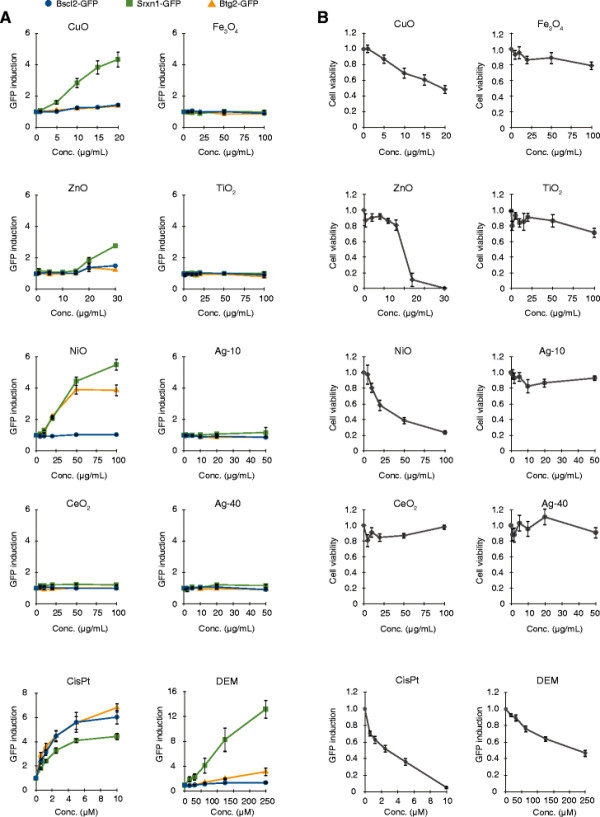
**The ToxTracker assay can identify genotoxic properties of NPs. (A)** The ToxTracker reporter cell lines Bscl2-GFP for DNA replication stress, Srxn1-GFP for oxidative stress and Btg2-GFP for p53-associated cellular stress were used to provide mechanistic insight into the biological damage that is induced by various metal-based NPs. Induction of the GFP reporters was determined by flow cytometry after 24 h exposure. The data show the mean of four independent experiments ± standard deviation of the mean. **(B)** Cytotoxicity of the tested NPs was determined by measuring the fraction of intact cells after 24 h exposure using flow cytometry.

Next, we exposed the ToxTracker cell lines to the eight different metal-based NPs for 24 h and recorded induction of the GFP reporters for DNA damage, oxidative stress and global cellular stress together with cell viability using a 96-well plate-based flow cytometer. Based on previous validation of the ToxTracker assay, a 1.5-fold induction of GFP expression was considered as cut-off for a positive test score [16]. A clear induction of the oxidative stress reporter (Srxn1-GFP) was observed for the CuO and NiO NPs (Figure 2A). Importantly, GFP reporter induction is exclusively determined in the intact, viable cells. Stem cells are generally very sensitive to cellular damage and in response rapidly activate their cellular signaling pathways and apoptotic programs. These properties make stem cells a highly sensitive system for detection of the primary toxic properties of chemicals and nanomaterials. The induction was observed for doses at which at least 40% of the cells were still viable and started from 10 μg/mL for the CuO and 25 μg/mL for the NiO NPs. The ZnO NPs showed induction of the oxidative stress reporter at concentrations from 20–30 μg/mL, but high levels of cytotoxicity (>75%) at these concentrations make the ToxTracker results inconclusive. None of the particles induced the DNA replication stress reporter (Bscl2-GFP), indicating that none of the tested NPs induced significant levels of replication-blocking DNA lesions. In addition to the oxidative stress reporter, the NiO NPs also induced the p53-dependent Btg2-GFP reporter, suggesting that the NiO NPs induced other types of biological damage to the cells. Exposure of the ToxTracker cells to CeO_2_, Fe_3_O_4_, TiO_2_ and Ag NPs did not result in any reporter activation (Figure 2A) nor induced any significant cytotoxicity at investigated doses (Figure 2B). The cell viability results based on flow cytometry were confirmed using the Alamar Blue cell viability assay (Additional file 1: Figure S4).

### Conventional DNA damage assays confirm the ToxTracker response to NPs

**Figure 3:**
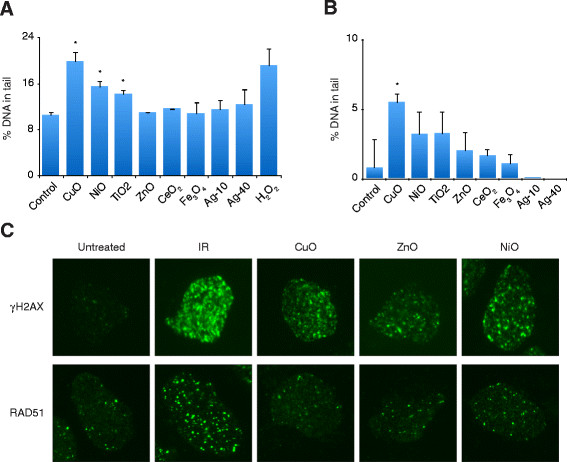
**Conventional DNA damage assays confirm the ToxTracker response.(A)** Induction of DNA strand breaks by the metal oxide NPs was determined by the comet assay under alkaline conditions. Wild type mES cells were exposed to NPs (20 μg/mL) for 4 h. H_2_O_2_ (10 μM for 10 min on ice) was used as positive control. DNA damage was quantified as percentage of DNA in the comet tail. Results are presented as mean ± standerd deviation of 3 independent experiments. **(B)** Induction of oxidative DNA lesions was determined by FPG comet. Wild type mES cells were exposed to NPs (20 μg/mL) for 4 h and results are expressed as net FPG sites. **(C)** Induction of γH_2_AX and RAD51 foci after 4 or 8 h exposure of mES cells to CuO (20 μg/mL), ZnO (30 μg/mL) and NiO (100 μg/mL) NPs as determined by immunocytochemistry. DSBs induction after 10 Gy IR was used as positive control.

In order to confirm the ToxTracker responses we tested the NPs in conventional DNA damage assays. First, we performed alkaline as well as FPG (Formamidopyrimidine DNA glycosylase) comet assay following mES cell exposure to the NPs at 20 μg/mL for 4 h (non-cytotoxic conditions). The alkaline comet assay is able to detect a wide range of DNA damage including strand breaks and alkaline labile sites (ALSs) whereas the FPG comet assay mainly detects oxidized purines [12]. Exposure to CuO and NiO NPs, as well as TiO_2_ NPs, gave significant tailing in the alkaline version of the comet assay (Figure 3A). The other NPs did not induce detectable DNA damage at these non-cytotoxic conditions. Furthermore, only CuO NPs caused an 5-fold induction of FPG sensitive sites, suggesting that oxidative stress is a main mechanism for CuO toxicity (Figure 3B). The ZnO also induced tailing in the alkaline comet assay at higher concentrations (Additional file 1: Figure S5). Additional analyses using the neutral comet assay, which is more indicative for double strand DNA breaks, showed no induction by CuO or NiO and only slight induction by ZnO (Additional file 1: Figure S5). The metal oxide NPs that showed a positive response in the ToxTracker were further investigated for their ability to induce γH_2_AX and RAD51 foci. The histone variant H_2_AX is rapidly phosphorylated at dsDNA breaks (DSB) but also at sites of chromatin relaxation and ssDNA breaks in mES cells [22]. RAD51 is a protein recruited to DSBs and plays an essential role in repair of DSBs by homologous recombination [23]. Exposure of mES cells to CuO, ZnO and NiO led to an increase in H_2_AX phosphorylation, although the levels were low compared with the control exposure to 10 Gy ionizing radiation (IR) (Figure 3C). In contrast, no RAD51 foci formation was observed after exposure to these NPs. Taken together, these results indicate that CuO induce ssDNA breaks and oxidative DNA lesions and thus confirms oxidative stress as a main mechanism as identified by the ToxTracker. NiO predominantly induce ssDNA breaks at non-cytotoxic conditions and possibly act via additional mechanisms that lead to p53 induction, as shown by ToxTracker. Clearly, the particles were not potent in inducing dsDNA breaks. ZnO only caused effects at doses that at later time points were highly cytotoxic.

### The toxicity of CuO is mediated via ions whereas that of NiO is particle-mediated

**Figure 4:**
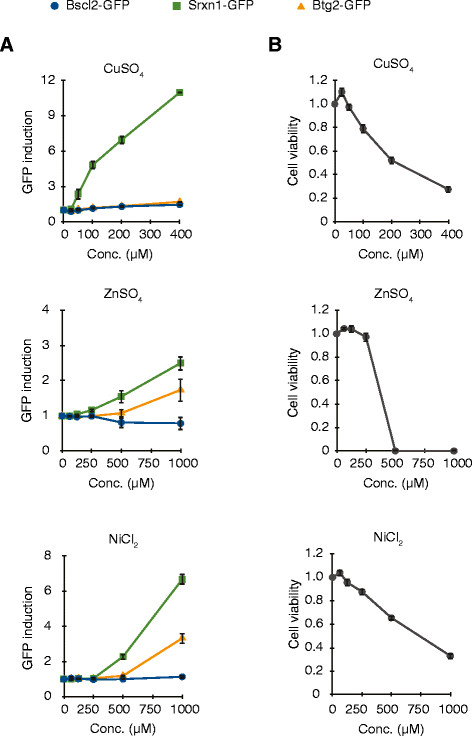
**Induction of the ToxTracker assay by metal ions.** mES cells were exposed to CuSO_4_ (25 – 1000 μM), ZnSO_4_ (50 – 1000 μM), NiCl_2_ (50 – 1000 μM) for 24 h. GFP induction and cell viability were determined with flow cytometry. Results are presented as mean ± standard deviation of three independent experiments.

Since the NPs that showed induction of the ToxTracker reporters (CuO, ZnO and NiO) dissolve to various extents in the cell culture medium, we investigated the ToxTracker response induced by dissolved metal ions from easily soluble metal salts at different concentrations. It should be noted that the metal dissolution from the oxide NPs occurs over time and that the cellular effect therefore is difficult to mimic. Furthermore, complexation between dissolved metal ions and cell medium components changes the metal speciation, reducing the free ion concentration with time. Dissolved Cu from CuSO_4_ (assuming 100% free Cu ions) induced the Srxn1-GFP oxidative stress reporter at doses starting from 50 μM (Figure 4A) without any effects on cell viability (Figure 4B). When compared with released concentrations of Cu from the CuO NPs (20 μg/L) after 0 h (8 μM) and 24 h (117 μM) it is evident that the dissolved Cu fraction from CuO NPs after 24 h was sufficient to induce oxidative stress. In the case of dissolved Zn from ZnSO_4_ (assuming 100% free Zn ions), the Srxn1-GFP reporter response was mainly induced at concentrations exceeding 500 μM and at these concentrations high cytotoxicity was observed making the ToxTracker results inconclusive (as for ZnO NPs). The particle effect was, however, pronounced for the NiO NPs compared to the response for dissolved Ni from NiCl_2_ (assuming 100% free Ni ions). For NiCl_2,_ the ToxTracker Srxn1-GFP reporter was induced at concentrations ≥500 μM and the Btg2-GFP reporter at concentrations of 1000 μM. Measured released concentrations from the NiO NPs after 24 h in cell medium were only minor (17 μM) compared to these concentrations and could therefore not explain the observed toxicity. A comparison between the concentrations used for NPs (CuO, NiO, ZnO) and their corresponding ions as well as the effect observed in ToxTracker is summarized in Table 2.

**Table 2:** The ability of NPs and their corresponding metal ions to induce the ToxTracker reporters

	**μg/mL**	**μM**	**μg metal/mL**	**ToxTracker induction**	**μg released metal/mL in cell medium**	**Is the released metal in cell medium likely to induce ToxTracker?**
**CuO NPs**	20	-	15,8	Yes	7,4	YES → ionic effect
**CuSO**_**4**_	-	50	3,2	Yes	-	
**NiO NPs**	20	-	15,6	Yes	1	NO → particle effect
**NiCl**_**2**_	-	250	14,7	No	-	
**ZnO NPs**	20	-	16,1	Yes (↑Tox)	15,7	Inconclusive due to high cytotoxicity
**ZnSO**_**4**_	-	500	32,7	Yes (↑Tox)	-	

### The ToxTracker assay responds to quartz particles but not to carbon-based materials

**Figure 5:**
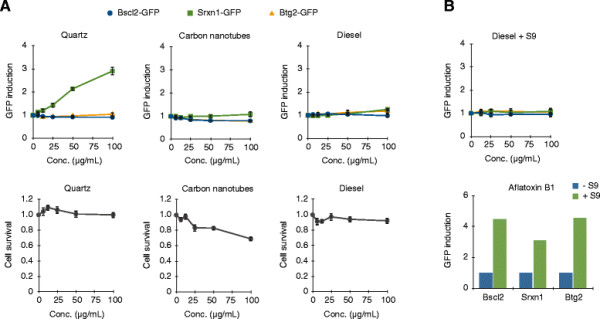
**ToxTracker induction by non**-**metal nanoparticles.(A)** The ToxTracker mES cells were exposed to quartz, multi-walled carbon nanotubes and diesel exhaust particles (6.25 – 100 μg/mL) for 24 h. GFP induction and cell viability were determined with flow cytometry. Results are presented as mean ± standard deviation of three independent experiments. **(B)** The ToxTracker mES cells were exposed to diesel exhaust particles in the presence of S9 rat liver extract for 3 h. GFP induction was determined after 24 h by flow cytometry. Results are presented as mean ± standard deviation of three independent experiments. Aflatoxin B1 was used as a positive control.

In order to benchmark the observed response to a particle control material we exposed the ToxTracker reporter cell lines to the carcinogenic quartz particles (DQ12). DQ12 is not a nanomaterial, but has often been used as a positive reactive particle control in both *in vitro* and *in vivo* studies on nanomaterials since it has high surface reactivity, inflammatory effects and induce oxidative DNA lesions at higher doses [24]-[26]. We also investigated whether the ToxTracker reporters were induced upon exposure to diesel particles (standard reference material SRM1650b) and carbon nanotubes (MWCNTs). Exposure to quartz particles clearly induced the Srxn1-GFP reporter at non-cytotoxic doses, starting from 50 μg/mL (Figure 5) supporting previous findings showing that ROS generation and more specifically hydroxyl radicals, play a major role for DQ12 induced genotoxicity [27]. On the other hand, no acellular ROS production was detected from the DQ12 particles (data not shown). In contrast, the MWCNTs and diesel particles did not induce the ToxTracker reporters. TEM images of mES cells exposed to MWCNTs indicated some uptake and there was also an increased side scatter shift analyzed by flow cytometry for both MWCNTs and diesel particles (Additional file 1: Figure S2 and Additional file 1: Figure S3). Thus, lack of uptake is not a likely explanation for the lack of effect in the ToxTracker reporters. Diesel exhaust particles consist of a mixture of polycyclic aromatic hydrocarbons (PAH), transition metals and quinones adsorbed on a carbon core that can lead to genotoxicity mainly via PAH-DNA bulky adduct formation and partly by oxidative DNA damage [28],[29]. Since PAHs require metabolic activation by cytochrome P450 enzymes in the liver and the lung before they become reactive, the effect of the diesel particles was also investigated in the presence of S9 rat liver extract. As a control for the activity of the S9 liver enzymes we treated the ToxTracker cells with the genotoxic compound aflatoxin B1 [16]. Treatment of mES cells with diesel particles in the presence of S9 did not lead to any reporter activation (Figure 5B). Thus, under the conditions tested in the present study, no pronounced genotoxicity/oxidative stress was observed for either the MWCNT or the diesel particles.

## Discussion

The fast expansion of manufacturing and use of nano-sized materials requires toxicity testing that should ideally be rapid, reliable, possible to use in a high-throughput manner and should provide information regarding the mechanisms of toxicity. These requirements are challenging to meet and will require development or implementation of novel tools. In this study we applied a novel fluorescence-based reporter assay for mechanism-based genotoxicity testing of various nanoparticles, in line with the 21^st^ century paradigm for nanomaterial testing which emphasizes the need for mechanism-based screening assays [2],[30]. The ToxTracker reporter assay has previously been shown to discriminate between compounds that can directly interact with DNA causing stalled replication forks (that may ultimately lead to mutations and genome instability) and compounds that act via oxidative stress and subsequent oxidative DNA lesions. The mES cells that are used in the ToxTracker assay are untransformed, proficient in all major DNA damage and cellular stress response pathways and shown in this study to efficiently engulf nanoparticles. Exposure of the ToxTracker cells to CuO and NiO induced the Srxn-1 reporter but not the Bscl2 reporter (Figure 2), indicating that the main mechanism of toxicity is oxidative stress rather than direct DNA binding and subsequent interference with DNA replication. This is also in line with the positive response in the alkaline comet assay (detecting ssDNA breaks and alkaline labile sites), the positive FPG comets for CuO, and the limited response in the neutral version of the assay as well as the lack of RAD51 foci formation (Figure 3). Identification of oxidative stress as primary mechanism of genotoxicity strongly decreases the carcinogenic hazard of these particles compared to agents that interact directly with DNA and interfere with DNA replication. The TiO_2_ NPs were positive in the comet assay but not in the ToxTracker. This might be explained by differences in sensitivity or possibly also that photocatalytic TiO_2_ NPs create additional strand breaks during the comet assay performance [12]. The Ag NPs included in the study showed no genotoxicity (comet assay and reporter cells) as well as no clear effect on the cell viability. Especially the lack of effects on cell viability after 24 h of the Ag-10 NPs was surprising considering the high cytotoxicity observed in BEAS-2B cells of these NPs [31].

In the present study, we carefully evaluated time-dependent dissolution/metal ion release from the metal (oxide) nanoparticles in cell medium by means of ICP-MS. In combination with experiments using soluble metal salts, we revealed that the effect observed for the CuO NPs could be explained by the released ions whereas the reporter activation (oxidative stress and p53 dependent stress) by NiO was related to the particles *per se* (Table 2). Indeed, our previous studies on lung epithelial cells suggested a high DNA damage potential of CuO and NiO NPs compared to other metal oxide NPs [17],[18] and the p53-dependent cellular stress response suggests additional reactivity of the NiO NPs compared to CuO. The uptake of the NiO was high as assessed by the TEM images. Likely, intracellular release of Ni ions could be important for the reporter response, which would be in line with the Ni-ion bioavailability theory describing that the carcinogenicity of a Ni-compound depends on the cellular uptake and subsequent availability of Ni in the cell nucleus [32]. Nickel ions from particles can be transported to the nucleus [33] and have been suggested to interact with DNA in a manner that causes silencing of tumor suppressor genes, *i.e*. an epigenetic effect [34]. Thus, even though we did not observe any NPs in the nucleus, metal ions from the NPs may still be present in the nucleus and interact with DNA, but in a manner that does not cause replication stress according to our results.

In order to benchmark the observed responses in the reporter assay after metal NP exposure, we compared responses to the carcinogenic quartz material called DQ12 that have previously been shown to cause oxidative stress and inflammation in various test systems [24]. It is therefore often used as a positive insoluble particle control in nanotoxicology studies [25]. In line with earlier reporters, DQ12 showed a positive response in the oxidative stress reporter cell line observed already at sub-toxic doses (Figure 5). Furthermore, both CuO and (especially) NiO showed intrinsic ROS generating ability (as observed in the DCFH acellular ROS generating assay), but this was not observed for DQ12. This supports our previous work suggesting that cellular interaction/lipid peroxidation is important for SiO_2_ NPs-induced toxicity [35]. ZnO did not produce acellular ROS and both ZnO and ZnSO_4_ only induced the oxidative stress reporter at highly cytotoxic doses (Figure 4B). This suggests that the reporter induction could be a secondary effect of the cytotoxicity in line with previous results that indicate that oxidative stress is not the primary cause of toxicity for ZnO [35],[36].

MWCNTs and diesel particles did not induce the ToxTracker reporters suggesting lack of direct DNA interaction and comparatively low oxidative stress potential. Previous studies have also reported low ROS generation of these materials, although a slight increase in DNA damage has been observed for the MWCNTs [17] and an increase in the mutation frequency following repeated lung cell exposure to diesel particles [37]. The ToxTracker reporter cell lines were thus not activated following exposure to low-reactive carbon-based materials. ToxTracker response following exposure to carbon particles with higher reactivity, such as Printex 90 [37], requires further testing.

In this study, we show the applicability of the ToxTracker assay for rapid screening of genotoxic properties of NPs. Other approaches have also been suggested for this purpose. Recently, Li and co-workers [38] proposed that DNA-binding assays can be useful in this regard and showed that NPs (size range 3–46 nm) with a high affinity for DNA strongly inhibited DNA replication (tested acellularly), whereas NPs with low affinity had no or minimal effect. However, such experimental acellular studies do not consider important factors such as the ability of the NPs to enter the nucleus and the fact that DNA is highly packed in mammalian cells. The likelihood for nuclear localization and DNA interaction depends on the NP size as well as its charge. Nabiev *et al*. demonstrated that green (2.1 nm) quantum dots (QDs) but not red ones (3.4 nm) entered the nucleus of THP-1 cells via nuclear pore complexes [8] and Conroy *et al*. [39] furthermore showed that QDs preferentially bind to the positively charged core histone proteins as opposed to the DNA. Size-dependent effect has also been reported for gold NPs as those with a very specific size of 1.4 nm have been shown to interact in a unique manner with the major grooves of DNA, which was suggested to be the reason for the high toxicity of these NPs [40]. Interestingly, only marginally smaller or larger particles showed significantly reduced toxicity [41]. Some studies have reported direct interaction of NPs with DNA in bacterial cells [42]-[44]. Possibly, DNA binding may be a more relevant mechanism in bacterial cells where DNA is more “naked” (histone-free) in contrast to mammalian cells where DNA is packed in nucleosomes and chromosomes. DNA may still be more exposed during spontaneously conformational fluctuations, in which DNA transiently unwraps the histone core [45] or at transcriptionally active sites. At present, the general importance of such mechanisms for the genotoxicity of nanomaterials for mammalian cells remains unclear.

## Conclusions

In this study, we have shown that the ToxTracker mES reporter cell assay can be applied as a rapid mechanism-based tool for assessing genotoxic effects of NPs. The assay is adapted to a 96-well plate format thus enabling medium/high throughput screening. CuO and NiO NPs caused a substantial reporter cell response that was related to oxidative stress rather than to direct interaction with DNA and stalled replication forks. NiO also induced the p53 dependent cellular stress reporter suggesting additional reactivity compared to CuO. Furthermore, the reporter cell induction appeared to be mediated by dissolved metal ions from CuO whereas the responses observed from NiO were related to the particles *per se*. The assay was validated for metal oxide NPs whereas the applicability for carbon based nanomaterials needs to be further investigated. Obtained results indicate that the ToxTracker reporter system can be used as a rapid mechanism-based tool for assessing genotoxicity of metal oxide NPs.

## Materials and methods

### Nanomaterials and soluble metal salts

Nanoparticles of CuO (20–40 nm), ZnO (20–200 nm), NiO (2–70 nm), CeO_2_ (4–30 nm), Fe_3_O_4_ (20–40 nm) and TiO_2_ (20–100 nm, mix of rutile and anatase) as well as multi-walled carbon nanotubes (MWCNTs, 100–200 nm in diameter and 3–7 μm in length) were obtained from Sigma Aldrich. TEM images of these particles have been provided in previous studies [17],[18]. Citrate coated Ag NPs (10 and 40 nm, 1 mg/mL dispersion in aqueous 2 mM sodium citrate) were purchased from NanoComposix, Inc (San Diego, CA), diesel exhaust particles (powder, SRM 1650b) were obtained from National Institute of Standards and Technology (Gaithersburg, MD, USA) and quartz particles (crystalline silica DQ12) were a kind gift from Prof. Roel Schins, Leibniz Research Institute for Environmental Medicine, Düsseldorf, Germany. Soluble metal salts of CuSO_4_, ZnSO_4_ and NiCl_2_ were purchased from Sigma Aldrich.

### Hydrodynamic particle size in cell medium

The size distribution in cell medium was investigated using dynamic light scattering (DLS) on an instrument employing photon cross correlation spectroscopy (PCCS) (NanoPhox, Sympatec, Germany). 20 μg/mL dispersions were prepared and analyzed directly after preparation. Single samples were measured three times at 25°C. Data from the unique measurements was integrated to produce a single distribution with the PCCS software. Standard latex samples (20 ± 2 nm) and blank samples were tested prior to analysis to ensure the accuracy of the measurements. The cell medium components resulted in a background contribution that was subtracted from the measured distribution for all nanomaterials investigated.

### Metal release in cell medium and cellular uptake of CuO

The amount of released metals in mES cell culture medium was determined by inductively coupled plasma optical emission spectroscopy, ICP-OES (Thermo Scientific iCAP 6500 duo). 20 μg/mL dispersions of CuO, ZnO, NiO, TiO_2_, CeO_2_, Fe_3_O_4_ NPs and of Ag-10 and Ag-40 NPs were prepared in complete cell culture medium. Metal release investigations were performed immediately after dispersion preparation (0 h) or kept at 37°C for 24 h after particle separation using a two-fold centrifugation procedure (30 min, 13000 rpm, 0°C). Successful particle separation was ensured using PCCS measurements. Total concentrations (limits of detection (LOD) in parenthesis) of Cu (0.3 μg/L), Zn (0.1 μg/L), Ni (0.4 μg/L), Ce (5 μg/L), Fe (0.3 μg/L), Ti (0.1 μg/L) and Ag (0.4 μg/L) in solution were analyzed using standard operational procedures with multiple standards for calibration (0, 100, 1000 μg/L) and triplicate measurements of each sample. Parallel measurements were performed on selected elements (Ag, Ti, Zn) for quality control using atomic absorption spectroscopy-graphite furnace, AAS-GF, (Perkin–Elmer Analyst 800). Mean metal concentrations of each element in solution are based on three replicate readings of each sample, independent of analytical method. Quality control samples were analyzed continuously throughout all analysis. All results are expressed as the released metal mass fraction of the total amount of exposed particles. The cellular uptake of CuO NPs was analyzed using AAS-GF as previously described [21].

### Acellular ROS generation

To measure acelullar ROS production, the 2',7'-dichlorofluorescein diacetate (DCFH-DA) assay was used. In brief, sodium hydroxide (0.01 M) was added to DCFH-DA to cleave the DA from the DCFH. The reaction was stopped by the addition of HBSS (Hank’s buffered salt solution). This solution was then incubated with the NPs in final concentrations of 10 and 50 μg/mL of NPs and 15 μM DCFH in 37°C for 30 min. Fluorescence was recorded every 5 min over 30 min (excitation 485 nm, emission 535 nm) using a plate reader (Tecan Infinite F200) at 37°C and ROS generation was calculated as mean slope per min and normalized to the blank. ROS generation was considered increased when the value was 1.5 or above.

### Preparation of nanoparticle dispersions

All powder particles were dispersed in cold mES culture medium by two times 10 min sonication at maximum power on ice (Bioruptor, Diagenode). The NP dispersions were diluted in warm BRL-conditioned mES culture medium, vortexed thoroughly and immediately added to the ToxTracker reporter cells. The Ag NPs were diluted directly in warm BRL-conditioned mES culture medium. NP dispersions were freshly prepared prior to exposure.

### mES cell culture and treatments

Culture of the ToxTracker mES cells was performed as described previously [16]. The mES cells were maintained in the presence of irradiated mouse embryonic fibroblasts as feeder cells in Knockout DMEM containing 10% fetal bovine serum, 2 mM GlutaMAX, 1 mM sodium pyruvate, 100 μM β-mercaptoethanol, and leukemia inhibitory factor (LIF). For NP analysis, cells were seeded 24 h prior to exposure on gelatin-coated plates using buffalo rat liver cell (BRL)-conditioned mES cell medium in the absence of feeder cells. Cells were continuously exposed for 24 h before GFP reporter analysis. The tested NP concentrations are based on cytotoxicity in mES cells after 24 h continuous exposure to a maximum test concentration at 50-75% cytotoxicity. In case of no observed cytotoxicity, a top concentration of 50 or 100 μg/mL was used. For analysis of diesel exhaust particles and compounds that require metabolic activation, cells were exposed for 3 h in the presence of 1% S9 rat liver extract in 3.2 mM KCl, 0.8 mM MgCl_2_, 0.5 mM glucose-6-phosphate and 0.4 mM NADP. After 3 h, the cells were washed with PBS and cultured for 24 h in BRL-conditioned medium without any nanomaterials.

### Cellular uptake and localization by TEM

For TEM analysis, wild type B4418 mES cells were exposed to 20 μg/mL CuO, 30 μg/mL ZnO, 100 μg/mL NiO, 100 μg/mL CeO_2_ NPs, 10 μg/mL Ag-10 and 20 μg/mL MWCNT for 24 h and subsequently fixed in 4% glutaraldehyde, rinsed in phosphate buffer (PB) and centrifuged. The pellets were then post fixed in 2% osmium tetroxide in 0.1 M PB, pH 7.4 at 4°C for 2 h, dehydrated in ethanol followed by acetone, and embedded in LX-112 (Ladd, Burlington, Vermont, USA). Ultrathin sections (approx. 60–80 nm) were cut by a Leica ultracut UCT (Leica, Wien, Austria) and contrasted with uranyl acetate followed by lead citrate and examined with in Tecnai 12 Spirit Bio TWIN transmission electron microscope (Fei company, Eindhoven, The Netherlands) at 100 kV. Digital images were captured by using a Veleta camera (Olympus Soft Imaging Solutions, GmbH, Münster, Germany).

### The ToxTracker assay

Bscl2-GFP, Srxn1-GFP and Btg2-GFP mES reporter cell lines were seeded in gelatin-coated 96-wells plates and exposed to various concentrations of NPs as described above. Induction of the GFP reporters was measured after 24 h continuous exposure using a 96-well Guava flow cytometer (Millipore) as described previously [16]. Simultaneously, cytotoxicity of the NPs was determined by measuring the concentration of intact cells after exposure using flow cytometry. All presented figures show the average GFP induction of at least three independent experiments. Error bars represent the standard error of the mean. The ToxTracker assay is considered positive above 1.5 fold increase in GFP signals. This limit is statistically based on 5 times the standard deviation of untreated controls and has been extensively validated in previous studies [16]. GFP induction levels at exposure concentration that induce >75% cytotoxicity after 24 h exposure are discarded for ToxTracker analysis.

### Alkaline and FPG Comet Assay

The mES cells were exposed to 20 μg/mL of nanoparticles for 4 h. The alkaline and FPG version of the comet assay was then performed as previously described [17] with some modifications. In short, cells were embedded in agarose and lysed for 1 h. For alkaline comet assay, alkaline unwinding and electrophoresis (29 V, 1.15 V/cm) was then performed for 40 and 30 min, respectively. For analysis of FPG-sensitive sites, following lysis cells were immersed (3 × 5 min) in FPG enzyme buffer and 30 μL of diluted (1:2500) FPG enzyme (kindly provided by Professor A. R. Collins, Department of Nutrition, School of Medicine, University of Oslo, Norway), or enzyme buffer, was added to each gel. Parafilm was placed on the slides, and incubation was performed in a humidity chamber at 37°C for 35 min. DNA unwinding and electrophoresis (1.15 V/cm) were then carried out for 30 and 20 min, respectively. The comets were examined on a fluorescence microscope (Leica DMLB and Leica Axioplan2, Houston, TX) with Comet Assay IV (Perceptive Instruments) and CometScore (TriTek). software. For each sample, 50 comets were evaluated in each experiment. The level of FPG-sites was obtained by subtracting the value of % tail obtained with no enzyme added from the value when FPG-enzyme was present.

### Immunocytochemistry

For detection of the DNA damage response after exposure to NPs, wild type B4418 mES cells were seeded on fibronectin-coated glass cover slips in BRL-conditioned mES cell medium. Cells were continuously exposed to 20 μg/mL CuO, 30 μg/mL ZnO, 100 μg/mL NiO NPs or to 10 Gy ionizing radiation as positive control. After 4 or 8 h, cells were fixed in 3% paraformaldehyde and stained for γH_2_AX or RAD51 as described previously [46]. RAD51 antibodies were a kind gift from Prof. R. Kanaar, rabbit anti-γH_2_AX and alexa488-labeled secondary antibodies were purchased from Millipore and Invitrogen, respectively. Images were taken with a Zeiss Axioplan2 microscope equipped with a Zeiss Axiocam MRm camera using either a Plan-NEOFLUAR 40x/1.30 or a 63x/1.25 objective.

## Abbreviations

AAS-GF: Atomic absorption spectroscopy-graphite furnace

ALS: Alkaline labile sites

ATR: Ataxia telangiectasia mutated and Rad3-related

AgNPs: Silver nanoparticles

DCFH-DA: Dichlorodihydrofluorescein diacetate

DLS: Dynamic light scattering

DSB: Double strand DNA breaks

FPG: Formamidopyrimidine DNA glycosylase

GFP: Green fluorescent protein

HBSS: Hank’s buffered salt solution

ICP-OES: Inductively coupled plasma optical emission spectroscopy

IR: Ionizing radiation

mES: Mouse embryonic stem cells

MWCNTs: Multi-walled carbon nanotubes

Nrf2: Nuclear factor erythroid 2-related factor 2

PAH: Polycyclic aromatic hydrocarbons

PCCS: Photon cross correlation spectroscopy

ROS: Reactive oxygen species

TEM: Transmission electron microscopy

QDs: Quantum dots

## Competing interests

The authors declare that they have no competing interests.

## Authors’ contribution

HLK initiated and planned the study together with GH, performed some of the experiments including alkaline and FPG comet assay, was involved in data interpretation and wrote the final version of the manuscript. ARG participated in the design of the study, carried out part of the cellular work, prepared samples for the particle characterization, was involved in data interpretation and drafted the manuscript. FC and CG carried out part of the cellular work including alkaline and neutral comet assay. IOW supervised the particle characterization, metal dissolution and ICP-OES analyses and their interpretations and was involved in manuscript writing. BF and HV were involved in data interpretation and in manuscript writing. GH initiated and planned the study together with HLK, performed most of the cellular work and wrote a first draft of the manuscript. All authors have given approval to the final version of the manuscript.

## Additional file

## Supplementary Material

Additional file 1: Supporting information. Click here for file
