# Supplementary material for: Mechanism-based genotoxicity screening of metal oxide nanoparticles using the ToxTracker panel of reporter cell lines
Source: Part Fibre Toxicol. 2014 Sep 2;11:41. doi: 10.1186/s12989-014-0041-9 (PMC4237954; doi:10.1186/s12989-014-0041-9)
Supplement: Additional file 1: — Supporting information. [file s12989-014-0041-9-S1.docx]

SUPPORTING INFORMATION


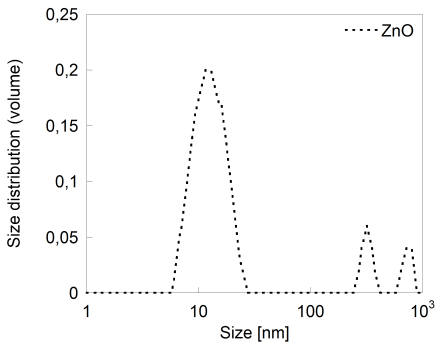

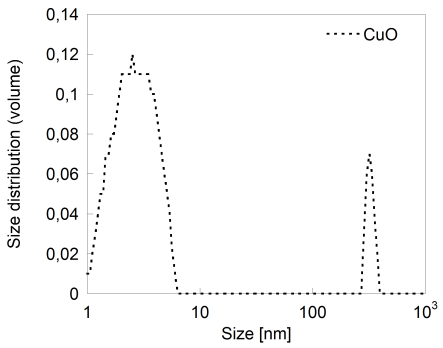


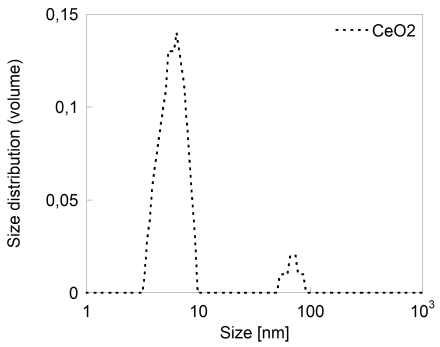

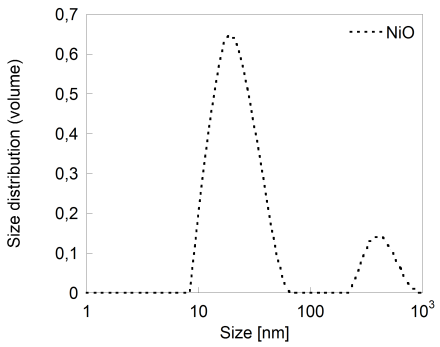


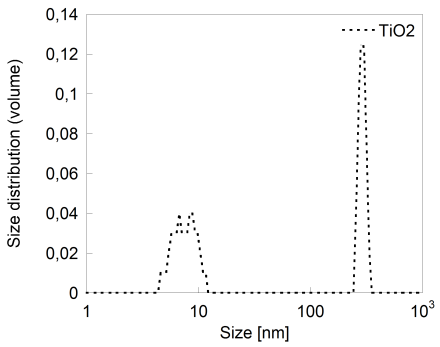

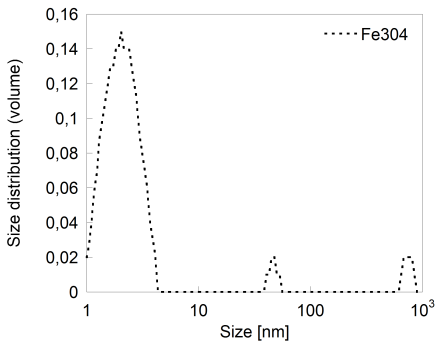


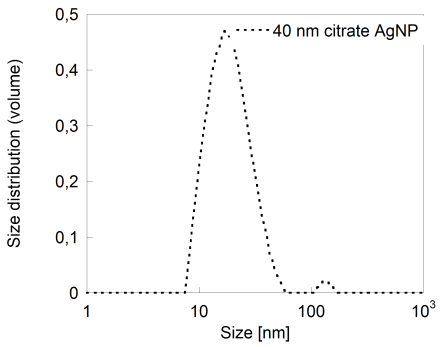

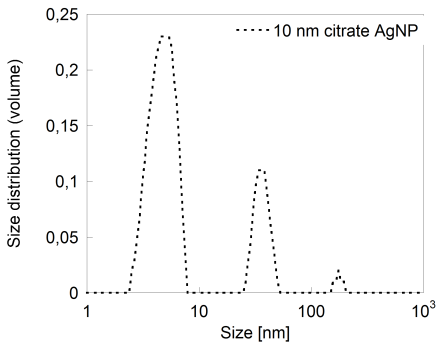


**Supplementary figure S1: PCCS histograms of the NPs in cell medium.**

**Supplementary figure S2: Cellular uptake of NPs by mES cells.** Uptake of all the NPs used in this study is determined by altered light scattering in flow cytometry. Cells were continuously exposed to the NPs for 24 h. (A) Scatter plots show analysis of 5000 cells exposed to the maximum NP concentration used in this study (see figure 2 and 5). (B) Quantification of changes in side-scatter (SSC) upon NP exposure. Box plots indicate 25 and 75 percentile, the minimum and maximum observed values and mean. Significance was determined in an unpaired two-sided t-test using GraphPad software. ^###^ p-value <0.0001.

**
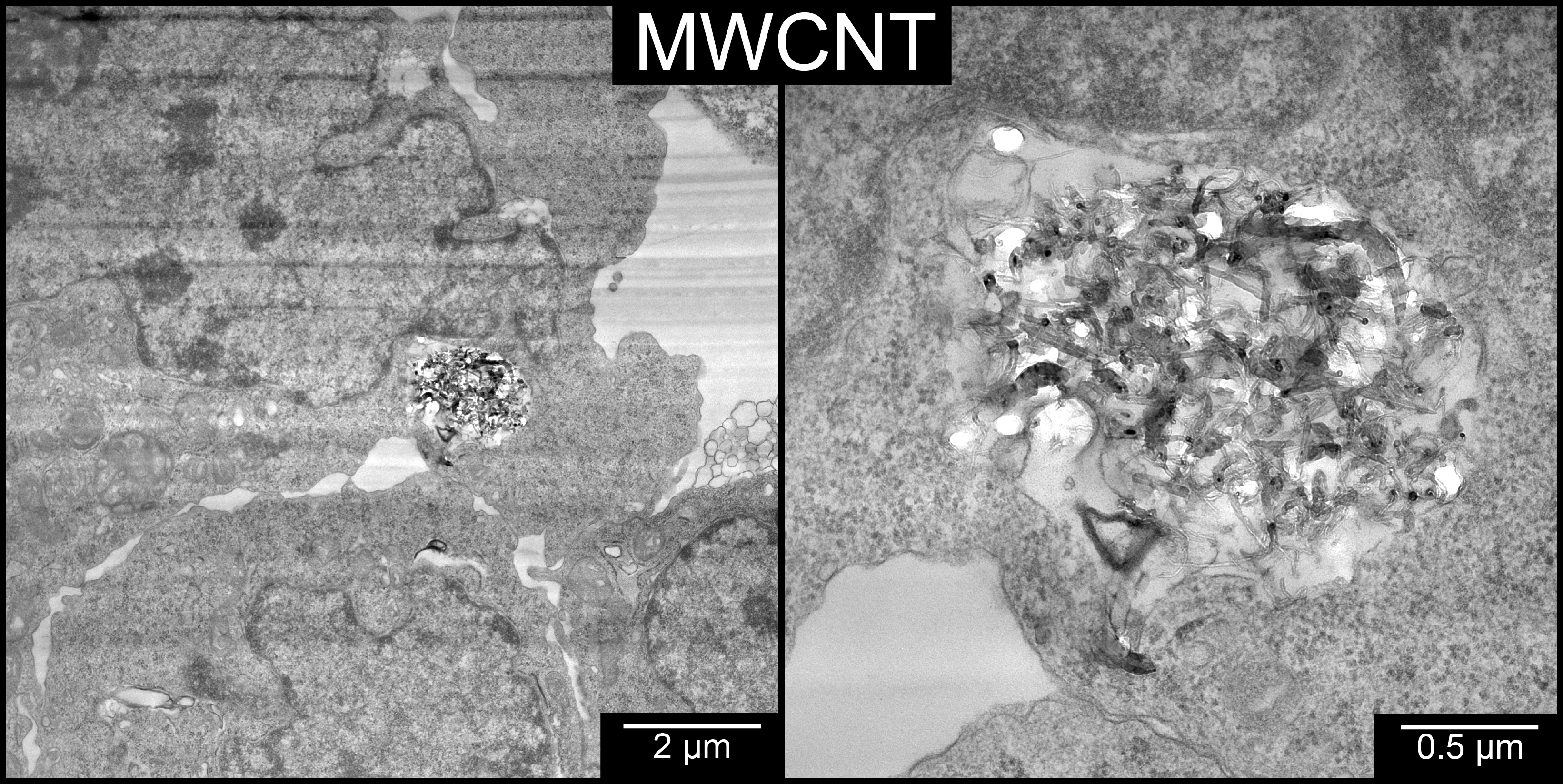
**

**Supplementary figure S3:** Cellular uptake of MWCNT by mES cells. Internalization of the NPs after 24 h exposure to 100 μg/mL MWCNT. Cellular uptake was determined by TEM.

**
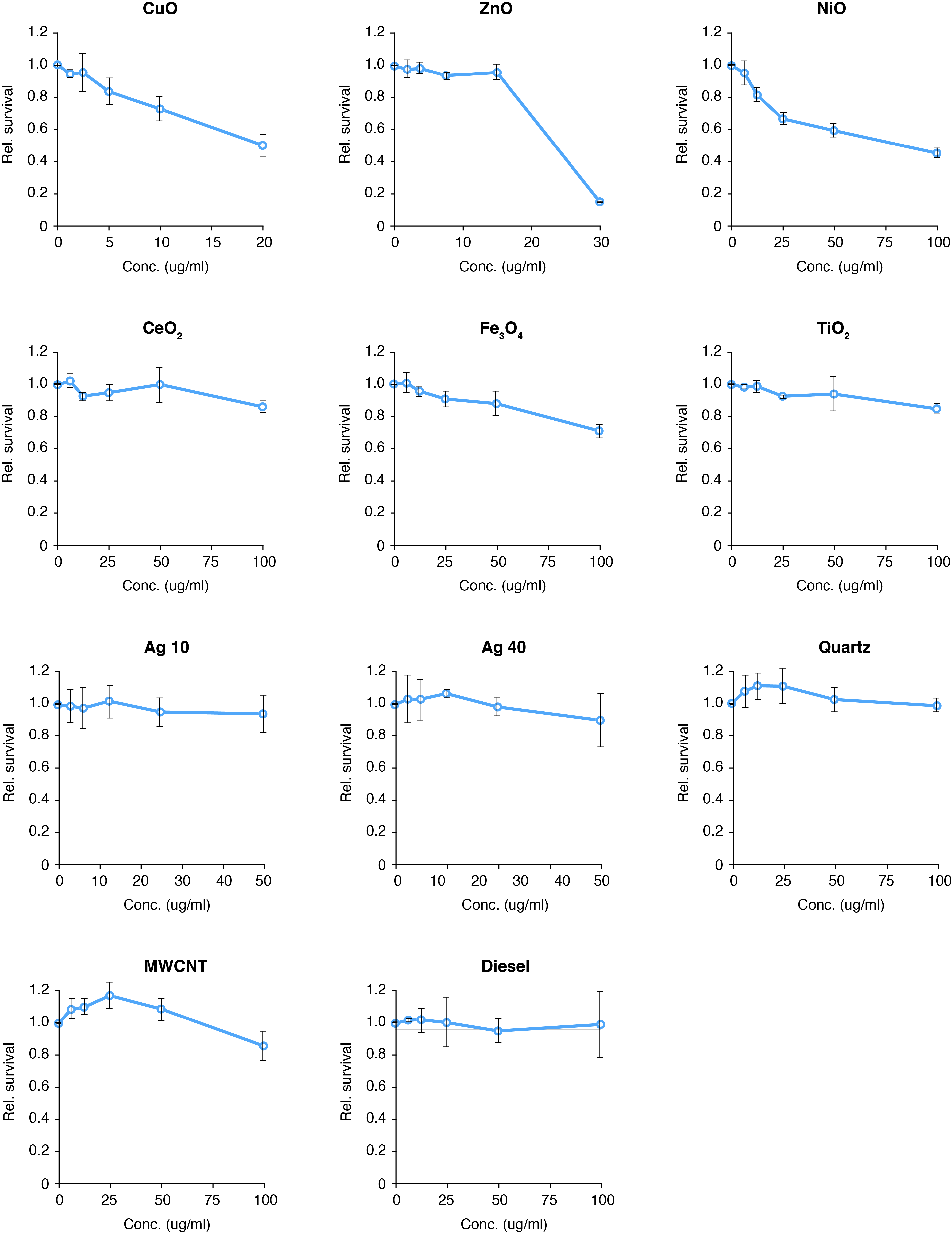
**

**Supplementary figure S4: Cytotoxicity of various NPs is mES cells.** Wild type mES cells were exposed to increasing concentrations of various NPs for 24 h. Cell survival was determined using the Alamar Blue cell viability stain. Shown graphs are a summary of three independent experiments. Error bars represent the standard deviation.

**
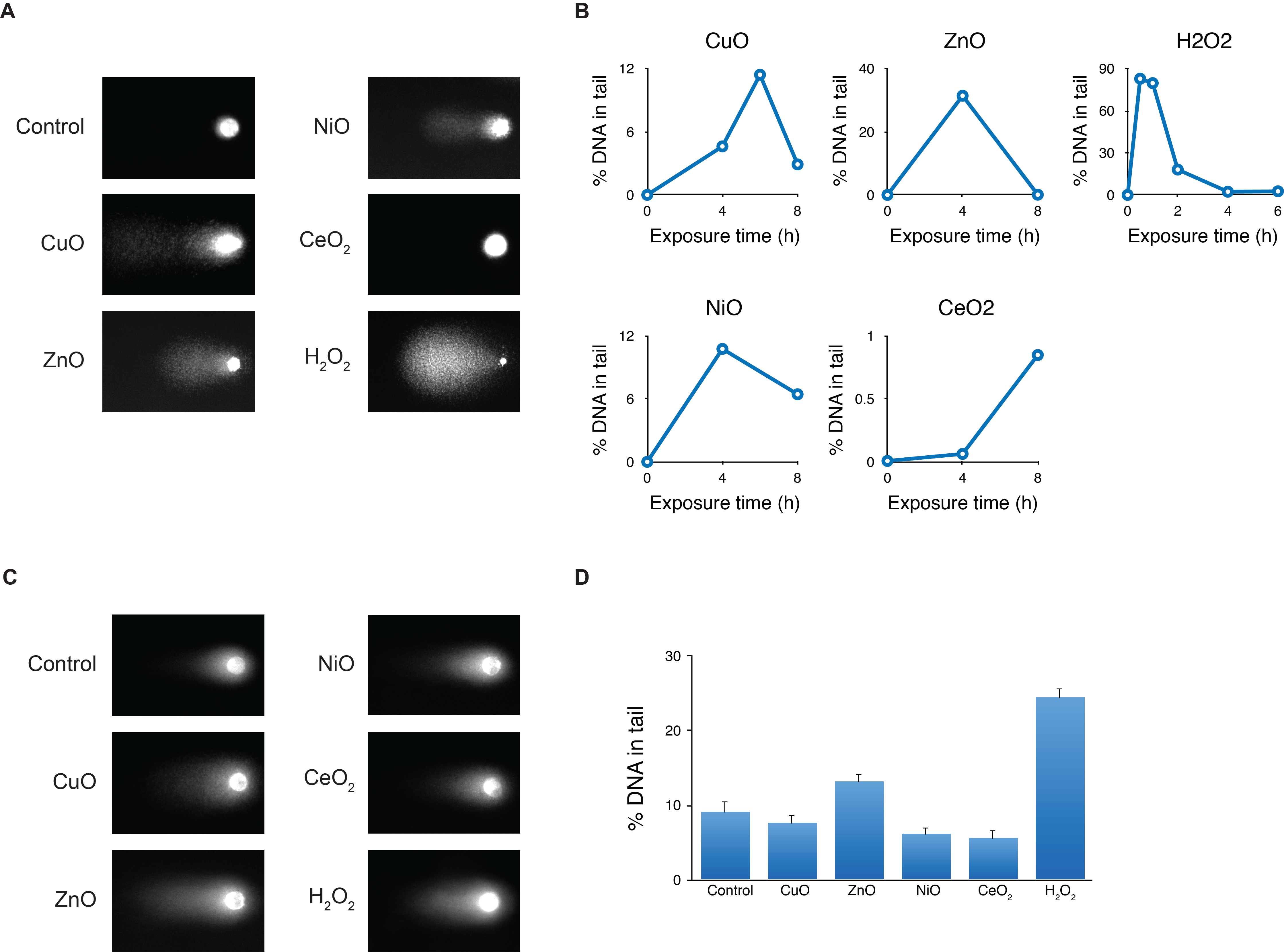
**

**Supplementary figure S5: Genotoxicity of nanoparticles by the comet assay.** (A) mES cells were exposed to equitoxic doses of CuO (20 µg/ml), ZnO (30 µg/ml), NiO (100 µg/ml) and CeO_2_ (100 µg/ml) NPs for 4 and 8 hours. Exposure to 0.5 mM H_2_O_2_ was used as positive control for the induction of DNA strand breaks. Induction of DNA strand breaks by the metal oxide NPs was determined using the alkaline comet assay. A representative picture is shown. (B) Quantification of the percentage of DNA in the comet tails. Comets were analyzed using a Zeiss Axioplan2 microscope CometScore software (TriTek). (C) Analysis of DNA damage induction upon exposure to various NPs by the Comet assay under neutral conditions. (D) Quantification of the percentage of DNA in the tails of the neutral comets.
